# Assessing psychopathology in bariatric surgery candidates: discriminant validity of the SCL-90-R and SCL-K-9 in a large sample of patients

**DOI:** 10.1007/s40519-020-01068-2

**Published:** 2020-11-23

**Authors:** Emanuela Bianciardi, Paolo Gentileschi, Cinzia Niolu, Marco Innamorati, Mariantonietta Fabbricatore, Lorenzo Maria Contini, Leonardo Procenesi, Alberto Siracusano, Claudio Imperatori

**Affiliations:** 1 Psychiatric Chair, Department of Systems Medicine, University of Rome “Tor Vergata”, Via Cracovia, 50, 00133 Rome, Italy; 2 Obesity Unit, Department of Surgery, University of Rome “Tor Vergata”, Rome, Italy; 3 Cognitive and Clinical Psychology Laboratory, Department of Human Science, European University of Rome, Rome, Italy

**Keywords:** Bariatric surgery, Binge eating disorder, Major depressive disorder, Obesity, Psychosocial assessment, SCL-90-R, SCL-K-9

## Abstract

**Purpose:**

Pre-surgical psychosocial evaluation of bariatric surgery (BS) patients should identify psychiatric issues that could worsen after surgery and those requiring additional ongoing intervention. In this view, the use of reliable, appropriate and concise evaluating instruments is of critical importance. The aim of the present study was to investigate the clinical utility of both the Symptom Checklist 90-Revised (SCL-90-R) and its brief unidimensional version, the so-called Symptom-Checklist-K-9 (SCL-K-9) in detecting the presence of psychiatric disorders among bariatric surgery (BS) candidates.

**Methods:**

Seven-hundred-and-ninety-eight BS candidates (563 women and 235 men; mean age: 44.15 ± 11.45) were enrolled in the present study. All participants underwent a full psychiatric interview and were administered the SCL-90-R.

**Results:**

Three-hundred-and-sixty-two patients (45.4%) met the criteria for a diagnosis of at least one psychiatric disorder and ninety-nine patients (12.4%) had psychiatric comorbidities. In the current sample, 219 patients (27.4%) met the criteria for binge eating disorders (BED), 158 (19.8%) met the criteria for major depressive disorder (MDD), and 67 (8.4%) met both criteria. A receiver operating characteristic (ROC) curves procedure showed that both the SCL-90-R and the SCL-K-9 satisfactorily categorize patients with any psychiatric disorder, both BED and MDD (area under the ROC curve ≥ 0.70, *p* < 0.001).

**Conclusion:**

Our results suggest that the SCL-90-R and the SCL-K-9 may represent first-level screening tests identifying at-risk patients, eligible for a more expensive or time-consuming clinical assessment.

**Level of evidence:**

Level V, cross-sectional, descriptive study.

**Electronic supplementary material:**

The online version of this article (10.1007/s40519-020-01068-2) contains supplementary material, which is available to authorized users.

## Introduction

Bariatric surgery (BS) represents a key factor among anti-obesity interventions. However, it is well recognized that weight loss is only one of the treatment goals because psychological and eating issues are prevalent before and after surgery [1, 2]. The most successful approach is, therefore, multidisciplinary [3].

According to the most recent meta-analysis [4] conducted on 65,363 individuals, 19% and 17% of BS candidates reported major depressive disorder (MDD) and binge eating disorder (BED) diagnoses, respectively. Using structured clinical interview, a lifetime prevalence of psychiatric disorders was also found in up to 70% of individuals [5]. However, psychiatric disorders do not necessarily prevent surgery, particularly when a proper treatment is developed [6]. Ideally, pre-surgical psychosocial evaluation should identify psychiatric issues that could worsen after surgery and those requiring additional ongoing intervention [7]. In this view, the use of reliable, appropriate and concise evaluating instruments is of critical importance.

Despite the historical lack of universal guidelines, the gold standard of the psychosocial behavioral pre-surgical evaluation is when psychiatrists and/or psychologists administer psychometric instruments while performing a clinical interview as well [8]. Though on the one hand, it was reported that the evaluation with self-report questionnaires may be vulnerable to several biases [9], on the other hand, the use of the clinical interview, such as the Structured Clinical Interview for the DSM (SCID), is more time-consuming, requiring preliminary training and a behavioral health specialist with expertise in the field of BS [10].

The Symptom Checklist 90-Revised (SCL-90-R) is a self-report questionnaire, assessing general psychopathology and clusters of psychiatric symptoms, which was suggested by the American Society for Metabolic and Bariatric Surgery as a valid screening measure to be used in the psychosocial evaluation [11]. The SCL-90-R demonstrated good internal consistency and validity among bariatric patients [12]. A recent study also showed that both the SCL-90-R and a brief unidimensional version, the so-called Symptom-Checklist-K-9 (SCL-K-9), were able to classify patients with overweight/obesity whether or not they showed significant binge eating symptoms [13].

To the best of our knowledge, no studies have investigated the clinical utility of both SCL-90-R and the SCL-K-9 in exploring the presence of psychiatric disorders among BS candidates. Making use of the receiver operating characteristic (ROC) curves procedure, we aimed to investigate the discriminant validity of the original SCL-90-R and the brief version SCL-K-9 in detecting psychiatric disorders. More specifically, we explored the clinical utility of both SCL-90-R and the SCL-K-9 in discriminating between: (1) BS candidates with or without any mental disorder, (2) patients with or without MDD, and (3) patients with or without BED, the most expected psychiatric disorders among this population [4].

## Materials and methods

This research is a part of a larger prospective study investigating the impact of psychiatric issues on BS candidates at our hospital [14]. The protocol includes the psychosocial assessment of consecutive BS candidates before surgery and subsequent follow-ups after six months, one, two and five years. The study was performed in accordance with the Helsinki declaration standards and was approved by the Institutional Ethic Review Committee of the University of Rome “Tor Vergata”; with all the participants providing written informed consent. The current data were collected at the time of the study entry (i.e. pre-operative psychosocial evaluation).

### Participants

Participants were 798 individuals (563 women and 235 men; mean age: 44.15 ± 11.45) referred to the Obesity Unit at the University of Rome “Tor Vergata”. The patients were enrolled according to the following criteria: age of 18 years and older; body mass index (BMI) of ≥ 30 kg/m^2^; negative history of cognitive impairment; negative history of substance and alcohol abuse; absence of any condition affecting the ability to complete the assessment.

### Measures

A trained senior psychiatrist with experiences in obesity and BS fields conducted a detailed psychiatric interview based on the full criteria of the last edition of the Diagnostic and Statistical Manual of Mental Disorders DSM-5; [15] to assess the presence of current psychiatric disorders. Patients’ weight and dieting history, motivation for seeking surgery, expectations concerning the surgical outcome, medical comorbidities and medication use were also recorded. All of the participants were also administered the Italian version of the SCL-90-R [16] that included the brief unidimensional version SCL-K-9 as discussed below.

The SCL-90-R [17] is a 90-item self-report on 5-point Likert scale (0–4) assessing general psychopathology and emotional distress in psychiatric, medical, and general population subjects. It examines 9 main psychopathological dimensions: somatic symptoms, interpersonal sensitivity, obsessive–compulsive behaviors, anxiety and depressive symptoms, hostility, phobic symptoms, paranoid tendencies and psychoticism. Furthermore, this scale provides a global severity index (GSI-90) which is proposed as an index of overall psychological distress, with higher scores reflecting higher levels of psychopathological distress as well as greater severity of self-reported symptoms [18]. The Cronbach’s α in the present sample was 0.98 for the GSI-90.

The SCL-K-9 is the brief unidimensional version of the SCL-90-R [19]. It is composed of the nine items of the SCL-90-R (#24, #28, #31, #34, #43, #57, #58, #75, #77) best representing (i.e., with the highest item-total correlation) all the original sub-scales of the SCL-90-R [19]. Satisfactory psychometric properties, including a one-factor structure, adequate internal consistency and convergent validity with psychopathology has been reported in both clinical [13] and non-clinical samples [19–21]. According to previous studies [13, 19, 21], the SCL-9-K was calculated on the basis of SCL-90-R questions. The Cronbach’s α in the present sample was 0.86 for the SCL-9-K total score (i.e., GSI-K-9).

### Statistical analyses

All analyses were performed with SPSS 18.0 statistical package for the social sciences (IBM, Armonk, NY, USA). In the present study, the ROC test procedures [22] have been performed to assess the performance of both the GSI-90 and the GSI-K-9 in categorizing, according to the DSM-5 diagnosis, individuals: (1) with and without any mental disorder, (2) with MDD (alone or in comorbidity) and without any mental disorder; (3) with BED (alone or in comorbidity) and without any mental disorder. An ROC curve is a two-dimensional depiction of test performance [23] and the area under the ROC curve (i.e., the probability that a randomly sampled respondent will be correctly assigned to the appropriate group) is considered the key outcome variable [24]. The AUC directly represents the overall accuracy of the instrument in categorizing a sample where values ≥ 0.70 are considered satisfactory [25]. The Youden Index [26] has been considered to classify the thresholds that maximize both sensitivity (i.e., the proportion of subjects who have the target condition and give positive test results) and specificity (i.e., the proportion of subjects who do not have the target condition and give negative test results). Furthermore, for sensitivity analyses, differences between patients according to the GSI-90 and the GSI-K-9 cut-off scores obtained from the first ROC curve (i.e., individuals with and without any mental disorders) were investigated using Chi-squared (*χ*^2^) tests and independent t-tests, respectively, for dichotomous and dimensional measures (supplementary material: Table S1, Table S2).

## Results

Patients had an average BMI of 44.18 kg/m^2^ (SD = 7.25) and an average age of 43.40 years (SD = 12.02: range 18–73). According to the standard BMI cut-off, there were 49 subjects with class I obesity (6.1%), 200 with class II obesity (25.1%), and 549 with class III obesity (68.8%).

Three-hundred-and-sixty-two patients (45.4%) met the criteria for a diagnosis of at least one psychiatric disorder and ninety-nine patients (12.4%) had a psychiatric comorbidity (i.e., BED and another psychiatric disorder). In the current sample, 219 (27.4%) met the criteria for BED, 158 (19.8%) met the criteria for MDD, and 67 (8.4%) who met both criteria. Detailed clinical and socio-demographic characteristics of the sample are reported in Table 1.

**Table 1:** Demographic and clinical data of patients

	Total (*N* = 798)
Variables	
Age—M ± SD	44.15 ± 11.45
Women—*N* (%)	563 (70.6)
Educational level (years)—M ± SD	11.29 ± 3.50
Unemployed—*N* (%)	254 (31.8)
Unmarried/not cohabitation—*N* (%)	389 (48.7)
Any medical comorbidity—*N* (%)	516 (64.7)
BMI—*M* (SD)	44.18 ± 7.24
BMI 30.0–34.9 kg/m^2^—*N* (%)	49 (6.1)
BMI 35.0–39.9 kg/m^2^—*N* (%)	200 (25.1)
BMI ≥ 40 kg/m^2^—*N* (%)	549 (68.8)
GSI-90—*M* (SD)	0.63 ± 0.53
GSI-9-K—*M* (SD)	0.72 ± 0.69
Any psychiatric disorder—*N* (%)	362 (45.4)
DSM-5 psychiatric diagnosis	
BED—*N* (%)	120 (15.0)
MDD—*N* (%)	91 (11.4)
Anxiety disorders—*N* (%)	24 (3.0)
Bipolar disorders—*N* (%)	6 (0.8)
OCD—*N* (%)	3 (0.4)
Psychotic disorders—*N* (%)	5 (0.6)
Personality disorders—*N* (%)	14 (1.8)
BED + MDD—*N* (%)	67 (8.4)
BED + anxiety disorders—*N* (%)	12 (1.5)
BED + bipolar disorders	4 (0.5)
BED + OCD	3 (0.4)
BED + psychotic disorders—*N* (%)	3 (0.4)
BED + personality disorders—*N* (%)	10 (1.3)

*BMI* body mass index, *GSI-90* global severity index of the Symptom Checklist-90-Revised, *GSI-9-K* global severity index of the Symptom Checklist-K-9, *DSM* diagnostic and statistical manual of mental disorders, *BED* binge eating disorders, *MDD* major depressive disorder, *OCD* obsessive–compulsive disorder

**Fig. 1:**
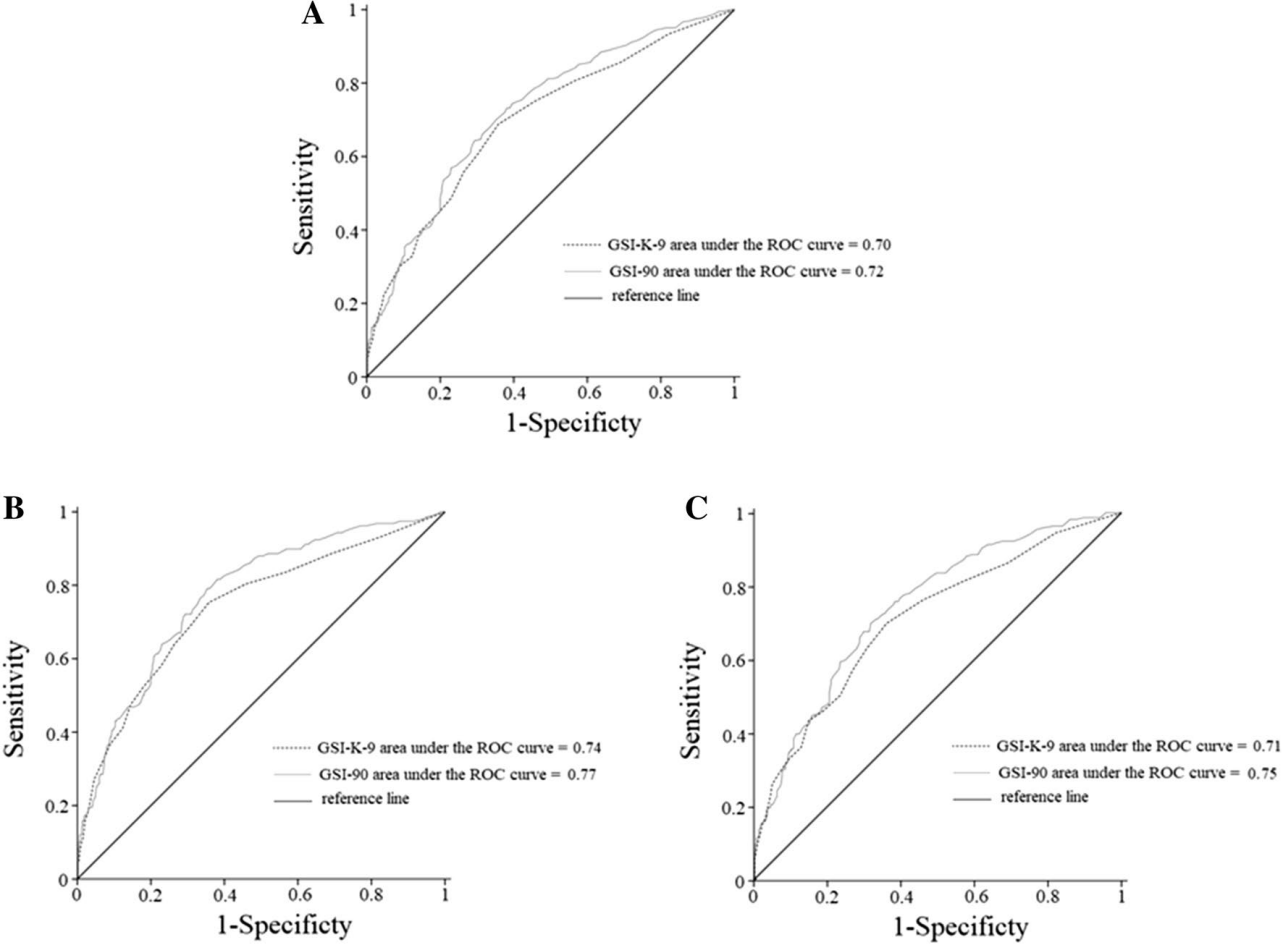
**a** ROC curve graph for the ability of GSI-9-K and GSI-90 to discriminate between patients with at least one psychiatric disorder (*N* = 362) vs patients without any psychiatric disorder (*N* = 436). **b** ROC curve graph for the ability of GSI-9-K and GSI-90 to discriminate between individuals who receive MDD diagnosis (*N* = 158) and those without any psychiatric disorder (*N* = 436). **c** ROC curve graph for the ability of GSI-9-K and GSI-90 to discriminate between individuals who receive BED diagnosis (*N* = 219) and those without any psychiatric disorder (*N* = 436). Abbreviation: *ROC* receiver operating characteristic, *GSI-9-K* global severity index of the Symptom Checklist-K-9; GSI-90 global severity index of the Symptom Checklist-90-Revised, *MDD* major depressive disorder, *BED* binge eating disorders

A ROC curve procedure indicated that both the GSI-9-K (area under the ROC curve = 0.70, 95% CI [0.66, 0.74], SE = 0.019, *p* < 0.001) and the GSI-90 (area under the ROC curve = 0.72, 95% CI [0.69, 0.76], SE = 0.018, *p* < 0.001) could categorize patients with at least one psychiatric disorder (*N* = 362) from those (*N* = 436) without any psychiatric disorder (Fig. 1a). Particularly a score of 0.50 or higher on the GSI-9-K (Youden index = 0.33) categorized individuals with a sensitivity of 0.69 (69% of all the patients with psychiatric disorders were correctly identified) and a specificity of 0.64 (36% of patients were incorrectly identified as having a psychiatric disorder). A score of 0.45 or higher on the GSI-90 (Youden index = 0.35) categorized individuals with a sensitivity of 0.73 (73% of all the patients with psychiatric disorders were correctly identified) and a specificity of 0.62 (38% of patients were incorrectly identified as having a psychiatric disorder).

When considering patients who receive a MDD diagnosis (*N* = 158) and patients without psychiatric diagnosis (*N* = 436), ROC curve procedure indicated satisfactory statistics (Fig. 1b) for both the GSI-9-K (area under the ROC curve = 0.74, 95% CI [0.69, 0.79], SE = 0.024, *p* < 0.001) and the GSI-90 (area under the ROC curve = 0.77, 95% CI [0.73, 0.81], SE = 0.022, *p* < 0.001). Particularly a score of 0.50 or higher on the GSI-9-K (Youden index = 0.40) categorized individuals with a sensitivity of 0.75 (75% of all the patients who receive an MDD diagnosis were correctly identified) and a specificity of 0.64 (36% of patients were incorrectly identified as having an MDD diagnosis). A score of 0.48 or higher on the GSI-90 (Youden index = 0.44) categorized individuals with a sensitivity of 0.79 (79% of all the patients who receive an MDD diagnosis were correctly identified) and a specificity of 0.65 (35% of patients were incorrectly identified as having an MDD diagnosis).

Finally, when considering patients who receive a BED diagnosis (*N* = 219) compared to patients with any psychiatric diagnosis (*N* = 436), an ROC curve procedure indicated satisfactory statistics (Fig. 1c) for both the GSI-9-K (area under the ROC curve = 0.71, 95% CI [0.67, 0.76], SE = 0.022, *p* < 0.001) and the GSI-90 (area under the ROC curve = 0.75, 95% CI [0.71, 0.79], SE = 0.020, *p* < 0.001). Particularly a score of 0.50 or higher on the GSI-9-K (Youden index = 0.34) categorized individuals with a sensitivity of 0.70 (70% of all the patients who receive a BED diagnosis were correctly identified) and a specificity of 0.64 (36% of patients were incorrectly identified as having BED diagnosis). A score of 0.52 or higher on the GSI-90 (Youden index = 0.38) categorized individuals with a sensitivity of 0.70 (70% of all the patients who receive a BED diagnosis were correctly identified) and a specificity of 0.69 (31% of patients were incorrectly identified as having a BED diagnosis).

## Discussion

The results of the current study confirmed that the rate of mental illness is considerable in BS candidates [5]. Indeed, 45.4% of patients were found to suffer from a psychiatric disorder. Furthermore, in accordance with previous studies [4, 27], we found that MDD and BED were the most prevalent disorders in this population.

However, our main aim was to explore the effectiveness of the SCL-90-R and the SCL-K-9 in identifying individuals with any psychiatric disorder and particularly, those suffering from MDD and BED, according to the clinical interview. We found a sensitivity of 73, 79 and 70% for the SCL-90-R, and 69, 75 and 70% for the SCL-K-9 suggesting that both self-reports may be valuable in detecting the most at-risk patients, through diagnosis during the pre-surgery evaluation. Although a psychiatric disorder per se is not a contraindication to surgery [28], it was recently highlighted that mental health service use increased, after surgery, especially among those with previous mental health service encounters, regardless of types of mental illness [29]. Specifically, both the SCL-90-R and the SCL-K-9 showed an adequate sensitivity in determining MDD and BED diagnoses that are prevalent and associated with negative sequalae in the bariatric setting. For example, self-harm and suicidal ideation may be symptoms of severe depression and increase after BS compared with the general population, especially in the long term [29]. Accordingly, a recent narrative review of metanalyses, emphasized that prior to surgery, there is the need to identify those individuals who are vulnerable to depression and self-harm [30]. Moreover, a powerful association between BS and subsequent depression was found [31]. On the basis of the bidirectional connection between depression and obesity [32], depression may lead to a poorer weight loss and an inadequate weight loss; moreover, reemergence of weight-related comorbidities may lead to depression [33, 34].

Actually, a link was also reported between BED and the clinical course of weight loss outcome after surgery, with pre-operatively BED individuals reporting a higher BMI in the long term after surgery [34]. Moreover, although after BS, rates of BED are quite low, patients may continue to fulfill the DSM-5 criteria for BED, except for the “unusually large” amount of food requirement, forming a “bariatric” BED subtype [35]. This emergent eating disorder is characterized by loss of control eating, leading to inadequate weight loss, weight regain and emotional distress [36–38].

Altogether, the SCL-90-R and the SCL-K-9 showed a satisfactory capacity to discern BS candidates with a psychiatric disorder, with MDD and with BED. As was previously reported, psychometric instruments were less effective in establishing a psychiatric diagnosis compared to a routine diagnostic interview [39]. In fact, the guidelines and the literature review suggest the use of a structured interview conducted by a mental health professional with specialized training in the bariatric field, as a gold-standard approach [40].

Nevertheless, the semi-structured clinical interview is expensive in terms of human resources, requires experienced staff, is time-consuming, and, therefore, may not be suitable for many clinical settings. Conversely, self-reports may be administered by various mental health professionals, such as psychologists, social workers, psychiatric nurses and psychiatrists. Therefore, we assume that the SCL-90-R or the SCL-K-9 may represent first-level screening tests, identifying the at-risk patients that will be eligible for a more expensive or time-consuming second interview. As a matter of fact, a successful approach could be devising flow charts for treatment care pathways in patients with psychiatric comorbidities or with high risk of comorbidities. This strategy may improve the use of specialized human resources that could be deeply involved in personalized second-level pre-surgery evaluation and perhaps provide additional behavioral interventions when needed [41]*.* Indeed, the important role of behavioral medicine should not be limited to the pre-operative period but needs an ongoing management of bariatric patients by appropriate pre-operative monitoring and treatments. Those additional interventions may strongly contribute to the efficacy of an interdisciplinary approach to obesity [42–44].

Furthermore, psychometric instruments provide replicable data for follow-up and research purposes.

Nevertheless, we recognize the limits of performing the SCL-90-R and the SCL-K-9. To start with, the clinical interview represents the most rigorous and effective evaluation instrument. The SCL-90-R and the SCL-K-9 are not able to distinguish between patients who suffer from psychiatric disorders and those currently affected by mental illness with symptoms in remission through treatment. Moreover, the SCLs do not assess substance and alcohol abuse and are vulnerable to the “impression management” phenomenon as discussed above. Despite this, we found that those instruments showed a satisfactory sensitivity to certain individuals affected by any mental illness, MDD and BED, and thus, they could be used as first-level screening tools. Exploring follow-up data, it would be stimulating to determine if SCLs were able to identify individuals at risk of adverse outcomes after surgery and, thus, to select patients who need close follow-up.

## Conclusion

In conclusion, our results demonstrated that the SCL-90-R and the SCL-K-9 were effective in recognizing MDD and BED that are the most prevalent psychiatric disorders in bariatric surgery candidates. These instruments may be used as first-level screening tests identifying at-risk patients, eligible for additional clinical assessment and behavioral intervention.

### What is already known on this subject?

Candidates for bariatric surgery should undergo a formal psychosocial evaluation performed by a mental health professional. The critical role of the psychiatric medicine is sustained particularly in the context of ongoing interdisciplinary management, assessing pre-operative and postoperative patient’s needs. The psychometric testing may be vulnerable to several biases, on the other hand, the use of structured clinical interview is more time-consuming and requires a behavioral health specialist with preliminary training.

### What this study adds?

We provide evidence about the effectiveness of the SCL-90-R and its brief version SCL-K-9 in identifying individuals with any psychiatric disorder and particularly, those suffering from MDD and BED, according to the clinical interview. The SCL-K-9 is composed of nine items of the original SCL-90-R. Thus, it is not extensive and time-consuming questionnaire. We demonstrated that the SCL-90-R or the SCL-K-9 may represent first-level screening tests in the bariatric population, identifying the at-risk patients that will be eligible for a more time-consuming second interview. After screening, the specialized behavioral team could be involved in personalized second-level pre-surgery evaluation and provide additional behavioral interventions when needed. Furthermore, these instruments may be used for follow-up and research purposes improving the interdisciplinary approach to obesity.

## Electronic supplementary material

Below is the link to the electronic supplementary material.

Supplementary Material 1 (19 kb)
