# Supplementary material for: Assessing psychopathology in bariatric surgery candidates: discriminant validity of the SCL-90-R and SCL-K-9 in a large sample of patients
Source: Eat Weight Disord. 2020 Nov 23;26(7):2211–8. doi: 10.1007/s40519-020-01068-2 (PMC8437842; doi:10.1007/s40519-020-01068-2)
Supplement: Supplementary file 1 — Supplementary Material 1 (19 kb) [file 40519_2020_1068_MOESM1_ESM.docx]

Differences between patients according to the GSI-9-K cut-off score (i.e., ≥ 0.50) are reported in supplementary table 1.

**Supplementary table 1.** Differences between patients according to the Symptom-Checklist-K-9 cut-off score (i.e., 0.50).

|  | **GSI-K-9** ≥ **0.50**  **(N = 405)** | **GSI-K-9** < **0.50**  **(N = 367)** | **Test** | ***p*** | |
| --- | --- | --- | --- | --- | --- |
| **Variables** |  |  |  |  |  |
| Age - M ± SD | 44.71 ± 11.56 | 43.58 ± 11.33 | t _796_= 1.40 | 0.163 |  |
| Women - N (%) | 313 (77.3) | 250 (63.6) | χ^2^= 17.94 | **<0.001** |  |
| Educational Level (years) - M ± SD | 11.08 ± 3.46 | 11.50 ± 3.53 | t _796_= -1.70 | 0.090 |  |
| Unemployed - N (%) | 146 (36.0) | 108 (27.5) | χ^2^= 6.75 | **0.009** |  |
| Unmarried/Not cohabitation - N (%) | 187 (46.2) | 202 (51.4) | χ^2^= 2.18 | 0.140 |  |
| Any Medical comorbidity - N (%) | 266 (67.0) | 250 (65.3) | χ^2^= 0.26 | 0.610 |  |
| BMI - *M (SD)* | 44.14 ± 7.42 | 44.24 ± 7.06 | t _796_= -0.21 | 0.838 |  |
| GSI-K-9 - *M (SD)* | 1.22 ± 0.63 | 0.20 ± 0.16 | t _458,66_= -32.23 | **<0.001** |  |
| Abbreviation: BMI = body mass index; GSI-90 = global severity index of the Symptom Checklist-90-Revised | | | | |  |

Compared to patients below the GSI subscale cut-off of the SCL-9-K, those above the cut-off were more frequently unemployed (36.0 % vs 27.5 %; χ^2^= 6.75; *p*= 0.009) and women (77.3 % vs 63.6 %; χ^2^= 17.94; *p*< 0.001). No other differences were detected, except those that were expected between the groups (i.e., GSI-K-9 total score).

Differences between patients according to the GSI-90 cut-off score (i.e., ≥ 0.45) are reported in supplementary table 2.

**Supplementary table 2.** Differences between patients according to the Symptom Checklist-90-Revised cut-off score (i.e., 0.45).

|  | **GSI-90** ≥ **0.45**  **(N = 431)** | **GSI-90** < **0.45**  **(N = 367)** | **Test** | ***p*** | |
| --- | --- | --- | --- | --- | --- |
| **Variables** |  |  |  |  |  |
| Age - M ± SD | 44.62 ± 11.88 | 43.60 ± 10.93 | t _796_= 1.26 | 0.209 |  |
| Women - N (%) | 320 (74.2) | 243 (66.2) | χ^2^= 6.16 | **0.013** |  |
| Educational Level (years) - M ± SD | 11.05 ± 3.43 | 11.57 ± 3.57 | t _796_= -2.12 | **0.034** |  |
| Unemployed - N (%) | 157 (36.4) | 97 (26.4) | χ^2^= 9.13 | **0.003** |  |
| Unmarried/Not cohabitation - N (%) | 199 (46.2) | 190 (51.8) | χ^2^= 2.49 | 0.115 |  |
| Any Medical comorbidity - N (%) | 286 (67.6) | 230 (64.4) | χ^2^= 0.88 | 0.349 |  |
| BMI - *M (SD)* | 44.13 ± 7.42 | 44.24 ± 7.06 | t _796_= -0.21 | 0.838 |  |
| GSI-90 - *M (SD)* | 0.98 ± 0.49 | 0.22 ± 0.13 | t _497,68_= 31.30 | **<0.001** |  |
| Abbreviation: BMI = body mass index; GSI-90 = global severity index of the Symptom Checklist-90-Revised | | | | |  |

Compared to patients below the GSI subscale cut-off of the SCL-90, those above the cut-off had lower educational level (11.05 ± 3.43 vs 11.57 ±3.57; t-test= -2.12; *p*= 0.034), were more frequently unemployed (36.4 % vs 26.4 %; χ^2^= 9.13; *p*= 0.003) and women (74.2 % vs 66.2 %; χ^2^= 6.16; *p*= 0.013). No other differences were detected, except differences that were expected between the groups (i.e., GSI-90 total score).
